# Correction to “HK2/VDAC1 Axis Regulates Malignant Progression of Esophageal Squamous Cell Carcinoma”

**DOI:** 10.1002/cam4.72175

**Published:** 2026-08-09

Dai, C. , J. Liu , Y. Guan , et al., “HK2/VDAC1 Axis Regulates Malignant Progression of Esophageal Squamous Cell Carcinoma,” Cancer Medicine 15, no. 7 (2026): e72102, 10.1002/cam4.72102.42438066 PMC13357688

The authors Yan Ma and Liping Su have been added as co‐corresponding authors. The updated correspondence section is as follows:

Correspondence:

Hongwei Pu (576250630@qq.com)

Yan Ma (522744476@qq.com)

Liping Su (1404720073@qq.com)

The online version of this article has been corrected accordingly.

We apologize for this error.

